# Supplementary material for: WordCommentsAnalyzer: A windows software tool for qualitative research
Source: F1000Res. 2018 Sep 4;7:536. Originally published 2018 May 3. [Version 2] doi: 10.12688/f1000research.14819.2 (PMC6137411; doi:10.12688/f1000research.14819.2)
Supplement: Supplementary file 3 [file f1000research-7-17614-s0002.tgz › b2a79496-4ada-47c4-9682-a99ce867836d.zip!/Eight Iranian Health Journals/Journal of Knowledge and Health_24.docx]

© Journal of Knowledge and Health. This work is licensed under CC BY 4.0 (https://creativecommons.org/licenses/by/4.0/).

Volume & Issue: دوره 7، شماره 2: 1391

Title: القای تمایز بن‌یاخته‌های مزانشیمی خون بندناف انسان به رده یاخته‌های شبه‌هپاتوسیتی در شرايط آزمایشگاهي

http://knh.shmu.ac.ir/index.php/site/article/view/79

مقدمه: بازسازی بافت‌ در محیط آزمایشگاه با استفاده از یاخته‌های بنیادی، یکی از موضوعات مورد علاقه محققان می‌باشد. بافت کبد نیز از این امر مستثنا نیست. هدف از این مطالعه، تولید یاخته‌های شبه‌هپاتوسیتی از بن‌یاخته‌های مزانشیمی خون بندناف می‌باشد. مواد و روش‌ها: بن‌ياخته‌هاي مزانشيمي، از خون بندناف (با کسب رضايت از مادران) جدا شدند و سپس ايمنوفنوتيپ بن‌ياخته‌هاي جداشده با فلوسايتومتري و توانايي تمايز آن‌ها با آزمون‌هاي تمايز در محيط آزمایشگاه بررسي شدند. درنهايت، بيان مارکرهاي اختصاصي کبد به‌واسطه واکنش‌هاي زنجيره‌اي پليمراز- معکوس (RT-PCR) آناليز شدند. نتایج: بن‌ياخته‌هاي مزانشیمی (Mesenchymal Stem Cells) جداشده از خون بندناف، توانايي تمايز به سويه مزودرمي را داشتند. چنانچه اين بن‌ياخته‌ها در شرايط مناسب کشت داده شوند، قادر به القای ژن‌هاي کبدي؛ مانند: آلبومين (Alb)، آلفافيتوپروتئين (AFP)، سيتوکراتين‌هاي 18 و 19 (18CK، 19CK) و آلفا-1-آنتي‌تريپسين (AA) هستند. همچنين در پي القاي تمايز به‌سمت هپاتوسيت‌ها، بن‌ياخته‌هاي مزانشيمي خون بندناف، پروتئين‌هاي آلبومين و آلفافيتوپروتئين را توليد مي‌کنند. نتيجه‌گیری: بن‌ياخته‌هاي مزانشيمي خون بندناف، توانايي تمايز به سويه‌ هپاتوسيتي را دارند؛ لذا ابزار مناسبي در درمان‌هاي ياخته‌اي با هدف بازسازي بافت کبد به‌شمار‌مي‌روند.

Title: نقش انتقال لکوسیت‌ها در القاي آسیب کبدی به‌دنبال ایسکمی– پرفیوژن مجدد کلیوی در موش سوری Inbred

http://knh.shmu.ac.ir/index.php/site/article/view/80

مقدمه: ایسکمی- پرفیوژن مجدد (Ischemia- Reperfusion) کلیوی، موجب آسیب رسیدن به ارگان‌های دور مانند کبد، مغز و ریه می‌شود. هدف از این مطالعه، بررسی نقش لکوسیت‌ها در القا آسیب کبدی، به‌دنبال ایسکمی- پرفیوژن مجدد کلیوی می‌باشد. مواد و روش‌ها: 18 سر موش‌ سوری به 2 گروه شامل گروه شاهد‌دهنده لکوسیت (Sham Donor) و گروه ایسکمی- پرفیوژن مجدد‌دهنده لکوسیت (JR Donor) (60 دقیقه بستن شریان‌های کلیوی دو طرف و سپس 3 ساعت پرفیوژن مجدد) تقسيم شدند. سپس لکوسیت‌ها از نمونه خون به‌دست‌آمده از قلب این دو گروه جداسازی شده و به گروه‌های دست‌نخورده (دو گروه 9 تایی) زیر منتقل شدند: موش‌های پذیرنده‌ای که لکوسیت‌ها را از گروه مداخله دریافت نمودند (IR Recipient) و موش‌های پذیرنده‌ای که لکوسیت‌ها را از گروه شاهد دریافت کردند (Sham Recipient). پس از 24 ساعت، حیوانات پذیرنده بیهوش شدند و نمونه‌هاي خون و كبد جمع‌آوری گردید. نتايج: غلظت آلانین آمینو ترانسفراز (ALT) و آسپارتات آمینو ترانسفراز (AST) خون و همچنین مالون دی آلدهید (MDA) کبدی گروه IR Recipient، افزایش معناداری نسبت‌به گروه Sham Recipient نشان دادند. در بافت‌شناسی نیز تغییر در فرم طبیعی سلول‌ها، واکوئولیزاسیون سیتوپلاسمی و انفیلتراسیون موضعی لکوسیت‌ها مشاهده گردید. نتیجه‌گیری: نتايج نشان می‌دهند که لکوسیت‌ها مي‌توانند یکی از عوامل آسیب کبدی به‌دنبال ایسکمی- پرفیوژن مجدد کلیوی باشند و این آسیب تا حدی ناشی از القاي استرس اکسیداتیو مي‌باشد.

Title: مقایسه عصاره رازیانه و ویتامین E بر مدت دیسمنوره اولیه

http://knh.shmu.ac.ir/index.php/site/article/view/81

مقدمه: دیسمنوره اولیه به‌عنوان درد لگنی در غیاب یک ضایعه پاتولوژیک مشخص در دوران قاعدگی تعریف می‌شود و یکی از مشکلات شایع زنان در سنین باروری می‌باشد. هدف این مطالعه، مقایسه اثر ویتامین E و عصاره رازیانه بر دیسمنوره اولیه مي‌باشد. مواد و روش‌ها: در اين كارآزمايي باليني يك‌سو كور، 65 دانشجوي دختر مبتلا به ديسمنوره اوليه متوسط تا شديد انتخاب، و به‌طور‌تصادفي در 3 گروه: عصاره رازیانه، ویتامین E و دارونما تقسیم شدند. گروه‌ها برای 2 ماه متوالی، درمان را از روز اول خونریزی به‌مدت 3 روز به مقدار 4 بار در روز به‌کار‌بردند. در گروه ويتامين E، كپسول 100 واحدي و براي گروه رازيانه از كپسول 46 ميلي‌گرمي عصاره رازيانه با نام تجاري فنالژين استفاده شد. نتايج: در گروه عصاره رازیانه، ميانگين مدت درد هم، در ماه اول و هم ماه دوم مصرف، نسبت‌به قبل کاهش معناداری نشان می‌دهد (001/0P<). اما در گروه ویتامین E، ميانگين مدت درد در دوره دوم بعد از شروع درمان كاهش يافته است (02/0P<). بین ميانگين مدت درد در 3 گروه در دوره اول تفاوت معناداري وجود دارد (033/0=P). نتیجه‌گیری: نتايج مطالعه نشان مي‌دهد كه عصاره رازيانه نسبت‌به ويتامين E، در طول مدت درد ديسمنوره اوليه مؤثرتر مي‌باشد.

Title: برآورد خطر ابتلا به سرطان‌های دستگاه گوارش فوقانی در افراد دارای سابقه فامیلی سرطان

http://knh.shmu.ac.ir/index.php/site/article/view/83

مقدمه: سرطان‌های دستگاه گوارش فوقانی، یکی از مهم‌ترین علل مرگ مبتلايان به سرطان در دنیا به‌شمار‌می‌روند. داشتن سابقه فامیلی، عامل خطرناك و جدی برای این نوع سرطان‌ها محسوب می‌شود، با وجود این، در ایران اطلاعات اندكي در این زمینه وجود دارد. هدف از این مطالعه، بررسی تأثیر سابقه فامیلی سرطان بر ابتلا به سرطان‌های دستگاه گوارش فوقانی است. مواد و روش‌ها: در این مطالعه مورد- شاهدی، 1010 مورد مبتلا به سرطان‌های دستگاه گوارش فوقانی و 1010 شاهد سالم، وارد مطالعه شدند. سابقه فامیلی سرطان در این افراد از فرم‌های استانداردی که پرسنل بهداشتی تکمیل كرده بود، استخراج شد. به‌منظور تعیین ارتباط سابقه فامیلی سرطان و خطر ابتلا به سرطان‌های دستگاه گوارش فوقانی، نسبت شانس تطبیق‌شده و حدود اطمینان 95% برآورد گردید. نتایج: سابقه فامیلی مثبت سرطان در 22% از موارد و 5/23% از شاهدها گزارش گردید (05/0<P). داشتن سابقه فامیلی سرطان معده و مری در بستگان درجه یک، به‌طور معناداری با ایجاد سرطان‌های دستگاه گوارش فوقانی در ارتباط بود و نسبت شانس تطبیق‌شده در این افراد 7/4 برآورد گردید (05/P<). نتیجه‌گیری: داشتن سابقه فامیلی سرطان، خطر ابتلا به سرطان‌های دستگاه گوارش فوقانی را افزایش می‌دهد. به همین دلیل، توصیه می‌شود بستگان افراد مبتلا به سرطان‌های دستگاه گوارش فوقانی، به‌عنوان گروه ارجح در برنامه‌های غربالگری مدنظر قرار گیرند.

Title: نقش اعتقادات بهداشتی، شکایات جسمانی و سلامت روانی بر کمردرد در پرستاران

http://knh.shmu.ac.ir/index.php/site/article/view/86

مقدمه: كمردرد یکی از شایع‌ترین ناراحتی‌ها در شغل پرستاری مي‌باشد كه ممكن است باعث ناتواني، محدودیت کاری، غيبت از كار و ايجاد آثار سوء اقتصادی زیادی شود. اين مطالعه با هدف تعيين نقش اعتقادات بهداشتی، شکایات جسمانی و سلامت روانی بر کمردرد در پرستاران اجرا شده است. مواد و روش‌ها: تحقیق حاضر یک مطالعه مقطعی است که بر روي کلیه پرسنل پرستاري (پرستار و بهيار) (246 نفر ) شاغل در بيمارستان‌هاي شاهرود در سال 87 انجام شد. از پرسش‌نامه CUPID برای جمع‌آوري داده‌ها استفاده گردید. براي تجزيه‌و‌تحليل داده‌ها نيز از آزمون‌های کای- دو، من- ویتنی و رگرسیون لجستیک استفاده شد. نتایج: در این مطالعه میانگین سن پرسنل پرستاری 7 ± 2/33 سال و ساعات کار در هفته 8 ± 5/47 ساعت بود. 69% از پرستاران بیش از 5 سال سابقه کار داشتند. در جامعه مورد مطالعه 59% (145 نفر) كمردرد را در 12 ماه گذشته گزارش کردند. نتایج نشان می‌دهد که بين کمردرد با سابقه كار پرستاري، كار كردن با دست‌هاي بالاتر از ارتفاع شانه، آشنایی با افراد مبتلا به کمردرد در خارج از محیط کار، شکایات جسمانی و اعتقاد به اینکه کمردرد به‌دلیل کار افراد ایجاد می‌شود، ارتباط معناداري وجود دارد (05/0>P). نتیجه‌گیری: علاوه‌بر عوامل جسمانی مرتبط با کار، اعتقادات بهداشتی و شکایات جسمانی نیز می‌توانند از عوامل خطر مرتبط با کمردرد محسوب شوند، که با مطالعات مشابه در این زمینه در کشورهای دیگر مطابقت دارد.

Title: بررسی عوامل مرتبط با درد ناحیه‌ گردن در کارمندان

http://knh.shmu.ac.ir/index.php/site/article/view/90

مقدمه: یکی از شایع‌ترین اختلالات اسکلتی- عضلانی مرتبط با کار در کارمندان اداری، درد ناحیه گردن مي‌باشد. هدف از انجام این مطالعه، تعیین شیوع درد ناحیه گردن و شناسایی برخی از عوامل مؤثر بر آن در بین کارمندان اداره‌های مختلف استان قم مي‌باشد. مواد و روش‌ها: این مطالعه نوعی بررسی مقطعی است که روی 1500 نفر از کارمندان ادارات دولتی مختلف در استان قم انجام شده است. روش نمونه‌گيري در اين مطالعه خوشه‌اي چندمرحله‌اي است. در این مطالعه از پرسش‌نامه‌ نوردیک برای تعیین درد ناحیه گردن استفاده شده است. نتایج: شیوع درد گردن در این مطالعه 1/20% با فاصله اطمینان (2/22- 1/18) برآورد شده است. عواملي كه ‌رابطه‌ معناداری با درد گردن داشتند شامل: جنس (011/0P=)، مدت‌زمان قرارگرفتن در وضعیت فیزیکی نامناسب (001/0P=)، نوع استخدام (001/0P<)، انجام کار با کامپیوتر (010/0P=)، سابقه تصادف با وسیله‌ی نقلیه (004/0P=)،‌ بلندشدن از پشت میز برای استراحت (005/0P=)، ‌شرایط محیط ‌کار ازنظر کارمند (016/0P=) و انجام فعالیت بدنی (009/0P=) بودند. نتیجه‌گیری: بسیاری از عوامل مرتبط با درد ناحيه‌ گردن قابل پیشگیری و کنترل مي‌باشند، لذا انجام برنامه‌ریزی‌های صحیح در راستای بهبود شرایط محیط کار مي‌تواند نقش مؤثری در پیشگیری و کاهش این اختلال داشته باشد.

Title: تأثير آموزش تن‌آرامی به مادران باردار بر کیفیت زندگی و شدت اندوه بعد از زايمان

http://knh.shmu.ac.ir/index.php/site/article/view/95

مقدمه: تغييرات جسمي و روحي ایجاد‌شده در دوران پس از زايمان، بر روان و كيفيت زندگي مادر تأثير مي‌گذارند. اندوه پس از زايمان ازجمله عوارضي است كه مي‌تواند بر سلامت جسمي و رواني او تأثير گذارد، بنابراین پیشگیری از آن می‌تواند در سلامت روانی خانواده مؤثر باشد. هدف این مطالعه بررسي تأثير آموزش تن‌آرامی به مادران باردار بر کیفیت زندگی و شدت اندوه پس از زايمان می‌باشد. مواد و روش‌ها: این مطالعه یک پژوهش کارآزمایی بالینی است که در آن 84 نفر از زنان باردار مراجعه‌کننده به بیمارستان‌های حافظ و شوشتری شهر شیراز، طی سال 1389 به‌صورت تصادفی به دوگروه آزمایش و شاهد تقسیم شدند. به گروه شاهد فقط مراقبت روتین و به گروه آزمایش علاوه‌بر آن، آموزش تن‌آرامی نيز ارائه گردید. درنهایت در روزهای اول و دهم بعد از زایمان، شدت اندوه با استفاده از ابزار خودسنجی افسردگی زونگ و کیفیت زندگی توسط مقیاس اختصاصی کیفیت زندگی سنجیده‌ شد. نتایج: دو گروه آزمایش و شاهد، ازنظر متغيرهاي دموگرافيك و مامايي تفاوت آماري معناداری نداشتند (05/0P>). نتایج پژوهش، اختلاف آماری معناداری را در شدت اندوه در گروه آزمایش و شاهد در روز اول و دهم نشان می‌دهد (05/0P<). همچنین در بازه زمانی ذکر‌شده، اختلاف معناداری در ابعاد احساس مادر خود و فرزندش در مقیاس کیفیت زندگی درگروه آزمایش نسبت‌به گروه شاهد وجود دارد (05/0P<).نتيجه‌گيري: براساس پژوهش حاضر، به‌نظر‌می‌رسد مداخله تن‌آرامی روشی بي‌خطر، مفيد و بدون هزينه در کاهش شدت اندوه و ارتقاي کیفیت زندگی پس از زايمان در مادران می‌باشد، بنابراين باتوجه‌به فوايد بسيار آن بر بهبود سلامت رواني مادر، انجام آن بعد از زايمان به کلیه مادران توصيه مي‌گردد

Title: تعيين ميزان دی‌اتیل‌هگزیل فتالئات (DEHP) موجود در وسايل مورد استفاده در همودياليز

http://knh.shmu.ac.ir/index.php/site/article/view/98

مقدمه: فتالئات‌ها در وسايل مورد استفاده در پزشكي ازقبیل فيلترها و لوله‎هاي رابط دياليزي يافت مي‎شوند. شواهد علمي بيانگر خطرات بهداشتي ناشي از مواجهه انسان با فتالئات‎ها هستند. در اين مطالعه ميزان دی‌اتیل‌هگزیل فتالئات (DEHP) موجود در وسايل مورد استفاده در همودیالیز (ساخت ایران) اندازه‌گیری شده است. مواد و روش‌ها: 10 نمونه لولة رابط مخصوص همودياليز ساخت ايران (5 نمونه مربوط‌به شركت ساخت وسايل پزشكي (سوپا) و 5 نمونه مربوط‌به سازمان تداركات پزشكي هلال‌‌‌احمر) به‌طورتصادفي انتخاب شدند. ميزان  DEHPموجود در هر نمونه لوله رابط به‌روش Gas chromatography - Mass spectrometry (GC-MS) اندازه‌گيري و ثبت شد. نتايج به‌دست‌آمده از هر 5 نمونه درمورد هر شرکت به‌صورت جداگانه باهم جمع و ميانگين غلظت DEHP موجود در هر نوع لوله رابط بر‌حسب میلی‌گرم در میلی‌لیتر گزارش گردید. نتایج: میانگین غلظت DEHP درمورد لوله‌های ساخت شرکت سوپا و هلال‌احمر به‌ترتیب 11/0 ± 36/1 و 11/0 ± 97/0 میلی‌گرم در میلی‌لیتر بودند. حداکثر و حداقل غلظت DEHP درمورد لوله‌های ساخت شرکت سوپا به‌ترتیب 47/1 و 21/1 میلی‌گرم در میلی‌لیتر و درمورد سازمان تداركات پزشكي هلال‌احمر به‌ترتیب 13/1 و 83/0 میلی‌گرم در میلی‌لیتر بودند. نتیجه‌گیری: می‌توان با جايگزين‎‌کردن محصولات پزشکی با مواد فاقد فتالئات يا فتالئات كمتر، مواجهه بيماران با فتالئات‌ها را کاهش داد
